# Geographic information analysis and web-based geoportals to explore malnutrition in Sub-Saharan Africa: a systematic review of approaches

**DOI:** 10.1186/1471-2458-14-1189

**Published:** 2014-11-20

**Authors:** Sabrina Marx, Revati Phalkey, Clara B Aranda-Jan, Jörn Profe, Rainer Sauerborn, Bernhard Höfle

**Affiliations:** Institute of Geography, Heidelberg University, Berliner Str. 48, 69120 Heidelberg, Germany; Institute of Public Health, Heidelberg University, Im Neuenheimer Feld 324, 69120 Heidelberg, Germany; Institute for Manufacturing, University of Cambridge, 17 Charles Babbage Road, Cambridge, CB3 0FS UK; Heidelberg Center for the Environment, Heidelberg University, Im Neuenheimer Feld 229, 69120 Heidelberg, Germany

**Keywords:** Geographic Information System (GIS), Spatial analysis, Web services, Malnutrition, Sub-Saharan Africa

## Abstract

**Background:**

Childhood malnutrition is a serious challenge in Sub-Saharan Africa (SSA) and a major underlying cause of death. It is the result of a dynamic and complex interaction between political, social, economic, environmental and other factors. As spatially oriented research has been established in health sciences in recent years, developments in Geographic Information Science (GIScience) provide beneficial tools to get an improved understanding of malnutrition.

**Methods:**

In order to assess the current state of knowledge regarding the use of geoinformation analyses for exploring malnutrition in SSA, a systematic literature review of peer-reviewed literature is conducted using Scopus, ISI Web of Science and PubMed. As a supplement to the review, we carry on to investigate the establishment of web-based geoportals for providing freely accessible malnutrition geodata to a broad community. Based on these findings, we identify current limitations and discuss how new developments in GIScience might help to overcome impending barriers.

**Results:**

563 articles are identified from the searches, from which a total of nine articles and eight geoportals meet inclusion criteria. The review suggests that the spatial dimension of malnutrition is analyzed most often at the regional and national level using geostatistical analysis methods. Therefore, heterogeneous geographic information at different spatial scales and from multiple sources is combined by applying geoinformation analysis methods such as spatial interpolation, aggregation and downscaling techniques. Geocoded malnutrition data from the Demographic and Health Survey Program are the most common information source to quantify the prevalence of malnutrition on a local scale and are frequently combined with regional data on climate, population, agriculture and/or infrastructure. Only aggregated geoinformation about malnutrition prevalence is freely accessible, mostly displayed via web map visualizations or downloadable map images. The lack of detailed geographic data at household and local level is a major limitation for an in-depth assessment of malnutrition and links to potential impact factors.

**Conclusions:**

We propose that the combination of malnutrition-related studies with most recent GIScience developments such as crowd-sourced geodata collection, (web-based) interoperable spatial health data infrastructures as well as (dynamic) information fusion approaches are beneficial to deepen the understanding of this complex phenomenon.

## Background

In recent years, an increasing interest on spatially oriented research has been established in health sciences [1–3]. The developments in Geographic Information Science (GIScience) such as interoperable and web-based Spatial Data Infrastructures (SDI) or highly accurate geospatial data acquisition techniques provide novel means to analyze and visualize the spatial dimension of several public health domains [1]. Geographic Information Systems (GIS) and geoinformation analysis methods are beneficial in identifying the most vulnerable parts of society in terms of malnutrition and living in poverty [4, 5]. Malnutrition is a serious challenge for the public-health system and “has been linked to a substantial increase in the risk of mortality and morbidity” [6]. Malnutrition refers to both undernutrition, which predominantly includes acute malnutrition (i.e. wasting), chronic malnutrition (i.e. stunting) and micronutrient malnutrition, as well as overnutrition or overweight [7]. It is the result of a complex and dynamic interaction between different factors such as health, socio-economic, political and environmental variables. In developing countries, prevalence of malnutrition in the form of undernutrition is still high with an estimation of about 850 million affected people between the years 2010 and 2012 [8]. In a global context, approximately 45% of the 6.6 million deaths of the under-five year old children in 2012 are caused by undernutrition [9]. Geographically, the majority of the undernutrition burden exists in Sub-Saharan Africa (SSA) and South-Central Asia [10]. SSA includes all African countries except for Northern Africa (Algeria, Egypt, Libya, Morocco, Tunisia and the Western Sahara) with the Sudan included in SSA [11]. In this area, about 40% (in 2011) of the children under five years suffer from chronic malnutrition [9].

As malnutrition is a complex phenomenon, the combination and joint temporal and spatial analysis of different data sources offer a new potential to get a better understanding. In this review, we refer to geographic information analysis as the “techniques and methods to enable the representation, description, measurement, comparison, and generation of spatial patterns” [12]. Since the Internet is becoming increasingly important in distributing and sharing information faster, the accessibility and availability of geoinformation and GIS software has increased, particularly for low- and middle-income countries [2]. However, despite the potential of GIS in health research, several barriers exist such as the lack of accurate spatial data, high costs and complexity of GIS software as well as privacy and confidentiality restrictions [2, 13, 14]. Hence, a systematic exploration of the current (web-based) applications of GIS and geoinformation analysis methods for studies on malnutrition is beneficial.

The primary objective of this Systematic Literature Review (SLR) is to determine how geoinformation analysis methods are applied for the investigation of malnutrition in SSA. We aim to identify which geodata, spatial levels and geoinformation methods are used as input to analyze malnutrition. Based on the literature review, further investigations are conducted with regard to the establishment of web-based geoportals for providing malnutrition data. Hence, a wider insight into the current state of malnutrition related research is given by taking recent developments in GIScience into account. Finally, we identify current limitations and discuss how new developments in GIScience might help to overcome impending barriers.

## Methods

An SLR, pioneered in the fields of medicine, is performed to summarize and qualitatively analyze research evidence on the spatial dimension of malnutrition studies in SSA. There are already some studies which adapt the medical guidelines and establish SLR in other research fields, e.g. computer science [15] or GIS-related research [16].

### Literature and geoportal search

We conduct a systematic search of three digital scientific journal databases: SCOPUS, ISI Web of Science and PubMed. Further peer-reviewed references as well as websites are added using a backward snowball method (pursuing references of references) [17]. Both approaches are done independently by two reviewers using the search strategy described in the protocol. All searches are limited to peer-reviewed articles in English published between January 2003 and July 2013. In light of the fact that spatially oriented research has been established in health research in recent years and is developing rapidly [2], articles published in 2003 and later are considered as being of prior relevance for the current state-of-the-art.

In order to identify relevant articles, firstly, a keyword search is performed within the peer-reviewed literature databases using the strategy outlined in Table 1. Within the three main concepts of geoinformation, public health and geographic focus of SSA, the terms are combined with a logical OR operator, whereas a logical AND operator is applied to join the three concepts.

**Table 1:** **Keywords for literature search classified by three comprehensive concepts**

Concept 1: geoinformation	Concept 2: public health	Concept 3: spatial focus
“remote sens*”	*nutrition OR wasting OR stunting OR undernourish*	*Africa*
GIS OR “geographic* information system*”	“food access” OR “food supply” OR “food production” OR “food *security”	
spatial OR “space-time” OR geospatial geostat*		

Only articles performing geoinformation analyses addressing malnutrition are defined to be relevant for this review. The geographic focus is restricted to SSA. Terms are searched as “MESH Terms” and all fields in PubMed, as topic field in Web of Knowledge and as abstract, title and keyword fields in SCOPUS. An example search performed in SCOPUS:



Duplicates resulting from the individual database query results are removed from the final list.

Secondly, further articles are extracted by hand-searching the reference lists of the identified full-text articles. The availability and accessibility of geodata are the prerequisites for geoinformation analyses. Thus, web-based geoportals that provide freely accessible geoinformation about malnutrition for the public are identified by using snowballing: The full-text articles are searched for web services as well as organizations. Based on these findings further relevant geoportals are acquired through a purposive Internet search.

### Data screening

After the database search, two reviewers independently carry out the selection of the articles in a standardized manner. Pre-identified inclusion and exclusion criteria with respect to the defined objectives are applied at each step to identify articles for full-text review: Articles that apply geoinformation analyses to explore malnutrition or food security in SSA are included in the review for title and abstract screening. However, at the last screening stage studies are excluded if they are either not dealing with malnutrition or indirectly address malnutrition e.g. as a potential consequence of food security. Since food security and malnutrition are closely linked, the criterion is not applied for the first (title screening) and second stage (abstract screening) due to the difficulty to differentiate them.

Two reviewers perform a first-stage title screening. If either of them decides that an article title is relevant, the article is included – otherwise the reference is excluded. In the second stage, all selected papers undergo an abstract screening. In this process, both reviewers have to agree to include a study according to the defined relevance criteria. Any disagreement is resolved by mutual agreement. After abstract screening, all included articles are reviewed for full-text.

### Data extraction and synthesis

All identified manuscripts are screened for study objectives, geoinformation analysis methods to explore malnutrition, relevant input geodata for malnutrition analysis, spatial scale of geodata and analysis level, study design (retrospective or prospective) as well as study location and analyzed timespan.

In a second step, several sub-categories are created to classify the extracted information. The geoinformation analysis methods are summarized into categories according to the type of analysis (e.g. spatial statistics and spatial modeling) used in the identified studies. The geodata are classified according to thematic information of the layers (e.g. population, infrastructure and agriculture) and spatial scale (household, local, regional or national). In terms of analysis level, it is distinguished between micro, meso and macro levels. Macro-level approaches are defined as analyses which look at broad trends such as the effects of climate change on public health across several countries. For example, at the macro level a country is treated as a single unit, whereas meso-level analyses look at spatial differences between sub regions within a country. Micro-level analyses operate on a local scale and consider individual or household-level factors. The identified web-based geoportals are analyzed as an additional source of information and are assessed for type of malnutrition indicators (classified as adult, maternal and child malnutrition), data format (web map, downloadable ready-to-use map, and downloadable GIS-ready data product) and spatial scale (regional and/or national).

## Results

**Figure 1:**
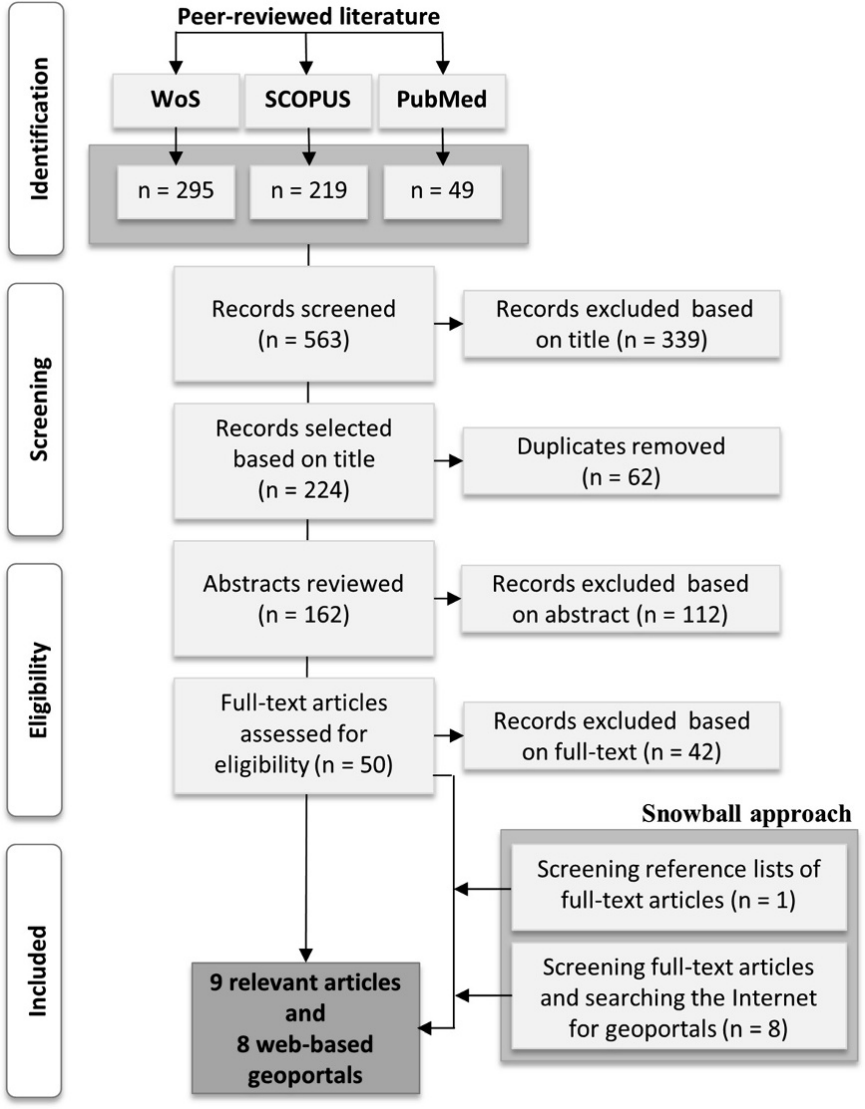
**Study flow for literature search to identify relevant articles and web-based geoportals.** Only English language articles published between 1st January and 31st July 2013 and freely accessible geoportals are considered.

A total of 563 references are identified of which 339 meet the relevant inclusion criteria in the first stage (title screening). 62 duplicates are excluded in step one. In the second stage we review 162 manuscripts for abstracts, of which 50 articles are identified for full-text review. 42 articles are excluded in the final step. One additional article is extracted by hand-searching the reference lists. Furthermore, eight web services are identified using the snowball approach. Thus, the SLR yields a total of 17 relevant hits, consisting of nine peer-reviewed papers and eight web-based geoportals (Figure 1). The extracted information from the full-text review of the peer-reviewed articles is summarized in Table 2. The main objective of the studies is to investigate the determinants (geographic, socioeconomic, environmental and/or biophysical) of malnutrition. Four studies [18–21] particularly focus on the effects of climatic factors on malnutrition.

**Table 2:** **Overview of the nine selected peer-reviewed articles**

Authors (year)	Study objectives	Spatial analysis method(s)	Geodata	Scale of geodata/level of analysis	Retro- or prospective (time span for analysis)	Geographic region
**Balk et al.**[23]	*Capture the effects of geographic and environmental variables on child hunger. Looking for causal relationships using micro-level data on a continental scale.*	- Spatial Statistics: *Simple ordinary least squares (OLS) regression analysis*	- Agriculture	Local and regional data/Micro- and meso-level analysis	Retrospective (1995–2004)	African, Asian and Latin American countries
			- Climate			
			- Health (+DHS*)			
			- Infrastructure			
			- Physiography			
			- Politics			
			- Population			
**Grace et al.**[19]	*Evaluate the relationship between climate variables and child malnutrition using a food security framework*	- Spatial Statistics: *Multi-level linear regression model*	- Climate	Household and local data/Micro-level analysis	Retrospective and Prospective (1990–2039)	Kenya
			- DHS*			
			- Livelihood			
			- Zones			
			- Population			
		- Spatial Interpolation: *Geostatistical interpolation using a moving window regression*				
**Jankowska et al.**[20]	*Examine and project climate and health trends in the African Sahel through the spatial coupling of climate data and health data in Mali.*	- Spatial Statistics: *Multivariate linear regression analysis*	- Climate	Local and regional data/Meso-level analysis	Prospective (1960–2039)	Mali
			- DHS*			
			- Livelihood			
			- Zones			
			- Physiography			
			- Population			
		- Spatial Interpolation: *Geostatistical interpolation using a moving window regression*				
**Kandala et al.**[25]	*Investigate the geographical and socioeconomic determinants of childhood undernutrition. Explore regional patterns of undernutrition.*	- Spatial Statistics: *Bayesian geo-additive regression model based on Markov priors*	- DHS*	Local data/Meso-level analysis	Retrospective (1992)	Malawi, Tanzania and Zambia
			- Socioeconomics			
**Liu et al.**[21]	*Spatially explicit assessment of current and future hotspots of food insecurity in SSA. Analyzing the impact of climate change on crop production.*	- Spatial Modeling: *Simulate dynamics of agricultural production*	- Climate	Local and regional data/Meso-level analysis	Prospective (1990–2030)	Sub-Saharan Africa
			- DHS*			
			- Economic			
			- Population			
		- Spatial Analysis: *Hotspot analysis*				
**Margai**[22]	*Discuss the multi-dimensional causes of food insecurity conditions, analyze the relation- ships between food insufficiency and nutritional health outcomes among children, and identify the demographic, socio-economic and environmental correlates of these conditions.*	- Spatial Analysis: *Road network distance analysis*	- Agriculture	Household and regional data/Meso-level analysis	Retrospective (1999)	Burkina Faso
		- Spatial Interpolation: *Kriging algorithm*	- DHS*			
		- Spatial Statistics/Statistical Methods: *Chi-square test, Logistic regression analysis*	- Infrastructure			
**Pawloski et al.**[26]	*Examine geographic relationships of nutritional status, including underweight, overweight and obesity among Kenyan mothers and children.*	- Spatial Statistics: Getis–Ord General G Statistics, Gi* Statistic	- DHS*	Local data/Meso-level analysis	Retrospective (2003/2006)	Kenya
**Rowhani et al.**[18]	*Present the influence of the climate-induced changes of ecosystem resources on malnutrition and armed conflict.*	- Spatial Statistics: *Logistic regression models*	- Agriculture	Local and regional data/Micro- and meso-level analysis	Retrospective (1946–2006)	Sudan, Ethiopia and Somalia
			- Economics			
			- Health			
			- Infrastructure			
			- Politics			
**Sherbinin**[24]	*Determine if, when controlling for income and the health conditions, biophysical and geographical variables help to explain variation in the rates of child malnutrition.*	- Spatial Statistics: *OLS Regression, Spatial Autocorrelation, Spatial Error (SE) model*	- Agriculture	Regional data/Meso-level analysis	Retrospective	Africa
			- Climate			
			- Economics			
			- Health (+DHS*)			
			- Infrastructure			
			- Physiography			
			- Population			

*The Demographic and Health Survey Program.

### Spatial dimension of malnutrition analyses

#### Method(s) for geographic information analysis of malnutrition in SSA

Methods for geoinformation analyses provide a wide range of different tools to explore the spatial dimension of malnutrition and range from descriptive maps displaying potential impact factors on malnutrition [18, 22] up to more complex depictions of the effects of social, economic and biophysical factors on malnutrition [23, 24]. They are largely applied to preprocess the input data: For example, spatial aggregation is used to combine datasets to one common spatial level [18]; buffering the road network can provide information about the access to transportation [22, 24] and at-point information from weather stations are spatially interpolated to create area-wide climate datasets [19, 20]. Furthermore, Liu et al. [21] employ a spatial modeling technique to simulate dynamics of agricultural production by integrating a GIS in an Environmental Policy Integrated Climate (EPIC) model. The results from the model are used as input for the prediction of future hunger hotspots in SSA.

The most common analyses to explore the spatial dimension of malnutrition in SSA are geospatial statistics e.g. spatial regression methods, which are applied by eight out of nine articles [18–20, 22–26]. Ordinary Least Squares (OLS) regression techniques are used to identify the geographic and biophysical correlates of malnutrition [23, 24]. Sherbinin [24] highlights that the effect of spatial autocorrelation on OLS regression models has to be accounted. Spatial autocorrelation refers to the occurrence of an event such as high malnutrition levels, which constrains or makes a similar event more probable in a neighboring unit. This effect is problematic for non-spatial statistical models. Thus, Sherbinin [24] performs a spatial error model that incorporates spatial effects through an error term. Kandala et al. [25] apply a Bayesian geo-additive regression model, which explicitly includes spatial information related to the observations. Another option to account for relationships across space is a multi-level regression analysis. This method enables Grace et al. [19] to predict malnutrition at household level while accounting for systematic unexplained variations at a coarser scale (meso level). Balk et al. [23] consider cluster specific spatial autocorrelation within the OLS regression but do not perform a more formal spatial regression. Likewise, other studies employ linear or logistic regression models to analyze the relationship between malnutrition and other spatial factors [18, 20] or to assess different risk levels of stunting [22], without accounting explicitly for spatial autocorrelation. Besides applying regression techniques, other geoinformation analysis methods help to improve the understanding of the spatial dimension of malnutrition. A geostatistical interpolation method (Kriging) is implemented by Margai [22] as a first step to evaluate the spatial prevalence of stunting. Thereby, a grid showing risk zones of childhood stunting is generated from the at-point health information. Pawloski et al. [26] identify statistically significant hotspots of malnutrition using the Getis–Ord Gi* statistics. The applied method determines spatial clusters of high or low malnutrition values. Liu et al. [21] extract problematic areas in terms of undernutrition by a combination of social, economic and biophysical factors in order to assess future hotspots of hunger.

#### Spatial scales of malnutrition analyses

The spatial analysis levels and the spatial scales of the underlying geodata, which might differ between the single input datasets, have to be taken into account when modeling and analyzing malnutrition. The level of analysis range from a micro-scale analysis [19] up to a continental-scale study [24]. It has to be noted that the spatial scale of the input geodata might differ from the spatial level of analysis. For example, Balk et al. [23] aim to link local data on a continental level. The selected studies, either work on one spatial analysis level [20, 21, 25], perform several geoinformation analyses on different spatial levels [18, 23] or consider two spatial scales in a multi-level analysis [19]. Six of the nine studies operate on the meso level [20–22, 24–26]. Balk et al. [23] and Rowhani et al. [18] employ a second analysis at the micro-level. None of the studies report a macro-scale analysis.

Once the spatial level of the analysis is chosen, the scale of the underlying geodata might have to be changed with different methods such as aggregation, down- and upscaling or interpolation techniques. This applies to studies working with input data on different spatial levels, which have to be linked. Five studies use geodata representing health and socio-economic parameters at household or district level in combination with regional datasets [18, 20–23]. The latter represent different potential impact factors on malnutrition such as climate, agriculture or politics (Table 2). Three studies [19, 25, 26] consider less impact factors but on a smaller scale (local datasets).

Every analysis level is associated with several strengths and shortcomings. A micro-level investigation based on household or individual data enables a more detailed assessment than meso- or macro-level analyses [22]. At this level of analysis other factors such as behavioral factors can be examined [23]. Furthermore, small-scale patterns as well as inter-regional differences can be considered [24, 25]. Due to the lack of data available, micro analysis based on individual, household or local datasets are limited to small parts of Africa [23]. At the sub-national and national scales, data availability is better. However, geographic variance within sub-national cannot be assessed based on these datasets [23, 25]. When coarser datasets are employed to analyze malnutrition at the micro level, the living conditions of the surveyed people may not be represented accurately [24]. At the meso level however, relationships which are not observable at the micro level can be analyzed [24]. This is due to the assumption that impact factors on malnutrition will be similar for people living closely together; thus, spatial differences may only occur at a larger scale [20]. Balk et al. [23] argue that household dynamics interact in a complex way and consequently are difficult to analyze at the micro level but become clearer through a regional-level lens. For example, in order to design policies for whole provinces, it can be helpful to summarize a problem at a meso level [23].

#### Relevant input geodata for malnutrition analyses

Nine different types of geodata are employed within the identified articles (Table 3). Geocoded health information is an important component in these studies. Furthermore, the studies attempt to explore links between malnutrition and climate, agriculture, infrastructure and (socio-) economics with a smaller emphasis on politics, livelihood zones and physiography. Most articles report concerns about the quality and availability of these data and identify it as a limitation of their study.

**Table 3:** **Thematic categories of geodata ranked by counts within the nine selected articles**

Thematic categories	Number of studies
**Health**	9
**Climate**	5
**Population**	5
**Agriculture**	4
**Infrastructure**	4
**(Socio)-Economics**	4
**Topography**	3
**Livelihood Zones**	2
**Politics**	1

##### Health

All selected studies employ health data to quantify the prevalence of malnutrition, most frequently data from the Demographic and Health Surveys (DHS) (eight out of nine studies). The DHS Program is funded by the United States Agency for International Development (USAID) and provides nationally representative data on population, health and nutrition for over 90 developing countries. Besides assessing health and nutrition parameters for women at child-bearing age, the questionnaire collects socioeconomic indicators for the entire household [25]. The most recent DHS surveys are geocoded (geographic latitude and longitude coordinates for each cluster of households recorded).

Common measurements of malnutrition based on DHS data include chronic malnutrition based on a height-for-age ratio (five articles) [19, 20, 22, 23, 25] and acute malnutrition based on a weight-for-age ratio (three articles) [20, 23, 24]. Body-Mass-Index (BMI) is used as an indicator for the nutrition status in two studies [21, 26] and level of anemia indicated by the amount of iron in the blood in one study [20].

Advantages of standard DHS surveys are that they have a large sample size and are typically conducted in regular five-year intervals. However, there are also challenges with georeferenced DHS data, e.g. the geographic coordinates are only recorded for the approximate population centroid of each DHS cluster [21] and the Global Positioning System (GPS) coordinate is displaced in order to maintain the confidentiality of survey respondents [27]. Rowhani et al. [18] do not employ DHS data but obtain an acute malnutrition indicator based on a weight-over-age ratio from the Complex Emergency Database (CE-DAT) [28]. Besides malnutrition data, other health data are used for geoinformation analyses e.g. to account for malaria [23, 24] or diarrhea [24].

##### Climate

Climate data are used in five studies. We can distinguish between historical climate data (five articles) [19–21, 23, 24] and projections of future changes in climate (three articles) [19–21]. For the conducted retrospective geoinformation analysis methods, raster datasets derived from satellite and ground-based meteorological stations are employed. Besides commonly used climate factors like precipitation and air temperature [19–21], other parameters such as the number of drought incidents can give a hint concerning the inter-annual variability in rainfall [24] or the length of the growing period [23]. Mentioned weaknesses of the used climate data are that in situ observations are very rare in most areas [19] and the data are often coarse in terms of their spatial resolution [21, 24]. Moreover, the datasets have an unknown level of error [24] and the uncertainties are high, especially for climate projections [20, 21].

##### Population

Historical and projected information about the population density are derived from gridded population data products. On the one hand, geospatial population data are used to analyze the impact of population changes [20, 21, 23] on malnutrition and, on the other hand, to restrict the study area by removing sparsely populated regions from consideration [19, 24].

##### Agriculture

The agricultural impact on malnutrition is represented by different agricultural variables in four references [18, 22–24]. Agricultural parameters can be derived from remote sensing data products [18, 23]. For example, the inter-annual variability of vegetation and ecosystem productivity is considered by Rowhani et al. [18] based on MODIS satellite data. Balk et al. [23] employ several agricultural variables such as the cropping or pasture intensity, the amount of cereal production or different soil constraints. Sherbinin [24] also uses agricultural constraints like soil, terrain and climate constrains. The latter two studies employ data products (e.g. Global Agro-Ecological Zones) from international organizations such as FAO, International Food Policy Research Institute (IFPRI) or International Institute for Applied Systems Analysis (IIASA) [23, 24]. Balk et al. [23] highlight that, despite being available in large numbers, most of the variables are imperfect proxies. There is a need to invest in more appropriate agricultural data to enable an analysis of the effect of agricultural productivity on malnutrition.

##### Infrastructure

Geodata on infrastructure are used to account for accessibility, which can serve as a measure for market access and trade. Furthermore, it is taken as a proxy for the level of urbanization and government service provision [24]. Balk et al. [23], Rowhani et al. [18] and Sherbinin [24] consider the road density, whereas Margai et al. [22] realize a distance analysis to account for the access to transportation. Besides using road networks, Balk et al. [23] add the distance to the nearest port as a further infrastructural variable to account for proximity to markets.

##### Livelihood zones

Another important factor for malnutrition are livelihood zones [20], which represent “geographical areas within which people share broadly the same patterns of access to food and income, and have the same access to markets” [29]. Grace et al. [19] and Jankowska et al. [20] include data on livelihood zones, provided by the Famine Early Warning Network (FEWS NET), in their analyses. The so-called livelihood zone maps are used to represent climatic sensitivity of households, for example [19].

### Web-based geoportals to explore malnutrition

Maps are a useful tool to visualize data within environment and health research, to interpret complex geographic phenomena and to identify spatial patterns. Therefore, they are an important tool for policy and decision making processes [30]. Furthermore, maps help to investigate the spatial distribution of malnutrition and to identify potential links to underlying causes. The use of the Internet has been firmly established to provide such digital geographic information and maps to everyone with internet access [31]. In this review we conduct further research based on the findings of the literature review and include eight web-based geoportals. The identified geoportals provide geographically linked information about malnutrition for SSA and partially also for other regions (Table 4). It is important to note that this is not a comprehensive list but it includes the most relevant portals identified by backward snowballing and a purposive Internet search. The aim of the identified databases is to monitor, evaluate and share information on the nutrition status at the national or regional level. This can serve as basis for trend analyses, impact briefings and policy recommendations. The review reveals that geoinformation about malnutrition, which is freely available to the public, is provided by international organizations such as the FAO [32] or the WHO [33, 34].

These geoinformation layers have global coverage but treat countries as a single unit. Further national malnutrition indicators for Africa are accessible through the Regional Strategic Analysis and Knowledge Support System (ReSAKSS) [35] facilitated by the IFPRI. In addition to national datasets, malnutrition indicators are stored at the regional level within the DHS Program’s Spatial Data Repository [36], funded by USAID and the Socioeconomic Data and Applications Center (SEDAC) [37], hosted by the Center for International Earth Science Information Network (CIESIN). The Complex Emergency Database (CE-DAT) [28], managed by the Centre for Research on the Epidemiology of Disasters (CRED), visualizes conducted malnutrition surveys for approximately 50 countries on a map. Over 3000 field surveys are stored in this database but only average values for mainly three malnutrition indicators can be accessed for each survey [28].

Available geoinformation are accessible mainly through interactive web maps, downloadable ready-to-use maps and downloadable GIS data products which may be used for further analysis. Geocoded primary datasets are rarely accessible via the identified, unrestricted parts of the geoportals. Only two geoportals provide GIS-ready regional malnutrition data [28, 36]. The downloadable DHS survey variables can also be visualized online in an interactive map on the DHS Program’s STATcompiler website [38]. Interactive web map services offer to display malnutrition data by interacting with the map such as panning, zooming or querying different map layers. In order to get a better understanding of malnutrition, the nutrition status is visualized along with potential impact factors [35] or nutrition policy and actions [34]. Furthermore, data can be displayed for different dates [28, 34, 38], which can additionally be used for a comparison between two timestamps [33]. For several reasons such as personal privacy only aggregated nutrition indicators are available. Nutrition information is obtained from different malnutrition indicators such as weight-for-height (wasting), height-for-age (stunting) and weight-for-age (underweight) and is available for adults and/or children. These datasets are collected within surveys or obtained from the government, albeit the availability varies widely between SSA countries.

**Table 4:** **Overview of selected, freely accessible web-based geoportals providing information about malnutrition in SSA**

Name	Organi-zation	Type of malnutrition data	Data format	Spatial scale of data
**Complex Emergency Database (CE-DAT),**[28]	CRED	Adult and child malnutrition indicators	- Web map	Regional and national
			- Downloadable ready-to-use map	
			- Downloadable GIS data product	
**Food Insecurity, Poverty and Environment Global GIS Database (FGGD),**[32]	FAO	Malnutrition indicators	- Web map	National
**Global Database on Body Mass Index,**[33]	WHO	BMI adults	- Web map	National
**Global database on the Implementation of Nutrition Action (GINA),**[34]	WHO	Malnutrition indicators	- Web map	National
**Socioeconomic Data and Applications Center (SEDAC),**[37]	CIESIN	Child malnutrition	- Web map	Regional and national
			- Downloadable ready-to-use map	
**The DHS Program: Spatial Data Repository,**[36]	USAID	Maternal and child malnutrition indicators	- Downloadable GIS data product	Regional and national
**The DHS Program STATcompiler,**[38]	USAID	Maternal and child malnutrition indicators	- Web map	Regional and national
**The Regional Strategic Analysis and Knowledge Support System (ReSAKSS),**[35]	IFPRI	Adult and child malnutrition indicators	- Web map	National

## Discussion

The literature reviewed herein has demonstrated that the spatial dimension of malnutrition is most frequently analyzed at the meso level using geostatistical analysis methods. Therefore, heterogeneous spatial data at different spatial scales and from multiple sources are combined by applying spatial interpolation, aggregation and downscaling techniques. Geospatial information about malnutrition is accessible to the public via web-based geoportals facilitated by international organizations such as the WHO or FAO. The malnutrition indicators are aggregated to the national or regional level and are visualized in maps. We rely on peer-reviewed literature as we assume that relevant scientific contributions are published in international journals - even if prior published in conference proceedings and reports. Furthermore, the reviewed articles include references to those reports and it is assumed that these findings contributed to the scientific progress published in these articles. Thus, this knowledge is included indirectly in this review. Additionally, relevant (scientific) data and results from international organization are identified and included by the geoportal search. Furthermore, an objective quality assessment is not performed due to missing validated standards for methodological GIS studies [16, 39]. The tools to assess the quality of observational studies were critically discussed by Shamliyan et al. [40]: Subjective judgments are very common and thus reduce the validity and reliability of quality assessments. In sum, this SLR relies on research-based evidences and qualitatively analyzes the potential of GIScience for malnutrition studies in SSA.

### Current challenges in the use of Geoinformation for research on malnutrition

The importance of the geospatial dimension in health research is asserted by several authors [1, 41, 42]. However, the absence of adequate geodata, especially at an individual level or for small areas as well as high uncertainties in some datasets (e.g. climate models), are identified as a major limitation in the reviewed literature. Problems with data quality and availability in low-resource countries are reported frequently and are not restricted to SSA [43–45]. Thus, there is a strong need for high quality data or at least with known accuracy in a fine temporal and spatial resolution. Mphatswe et al. [46] assert that “reliable and accurate public health information is essential for monitoring health and for evaluating and improving the delivery of health-care services and programs”. Furthermore, current geoinformation analysis approaches as identified in this review need to be adapted, extended or newly developed to take care of heterogeneous data quality and different spatial scales in the process of data and geoinformation fusion [47, 48].

Not all available malnutrition-related data are accessible. Data sharing between different administrative levels is described as challenging [41]. Web-based GIS applications provide the means to manage and distribute geospatial data efficiently and can also be used to deal with health-related data as it is shown in this review. However, web-based health applications still “show uncertainties regarding data sharing and interoperability” [41]. Moreno-Sanchez et al. [49] suggest that open source software and open specifications can meet the challenges “to the creation and deployment of interoperable cross-border health spatial-information systems”. User-friendly, interoperable (web-based) health SDI present advantages for malnutrition studies in which geodata from various sources have to be combined frequently. Further research is needed to augment SDIs on all administrative levels with spatial needs of health applications.

A significant concern about gathering spatially referenced health data are privacy and confidentiality restrictions - a general barrier for the adoption of GIS in health science [14]. Only aggregated malnutrition information is accessible for the public via web-based geoportals. For scientific interests data from the DHS Program are accessible for household clusters, but coordinates are displaced to protect the identity of the individual [27]. Granell et al. [41] report that “most health-related data are natively aggregated. For example, public health agencies report yearly on the number and cases at a district, city or even regional level”. Privacy issues are not to be underestimated in health related studies and new methods have to be developed to protect the privacy of survey respondents while allowing for exploring spatial relationships in detail [2].

## Conclusions

The SLR reveals that beyond mapping malnutrition prevalence, ordinary regression models as well as advanced spatial statistics are employed to explore the impact of environmental and other geographic factors on malnutrition in SSA. Therefore, heterogeneous spatial data at different spatial scales and from multiple sources are combined by applying spatial interpolation, aggregation and downscaling techniques. Aggregated geographic information (mostly web maps and downloadable map images) about malnutrition is already freely accessible via web-based geoportals. However, parts of SSA are not covered at all or are investigated at a regional or national level. Recent developments in GIScience demonstrate the potential to overcome current limitations such as the lack of accurate small-scale data, privacy issues and restrictions on sharing geocoded malnutrition data as well as the high costs and complexity of GIS applications. In future, the combination of new geospatial datasets and GIS methods such as crowd-sourced geodata collection and (dynamic) information fusion approaches, with malnutrition-related studies could be beneficial to deepen the understanding of this complex phenomenon in SSA. Increased data availability and accessibility via (web-based) interoperable health Spatial Data Infrastructures (SDI) provide the basis to explore malnutrition further. Such research could provide new methods for detailed multi-sensor earth observation and health-geoinformation analysis. Furthermore, health agencies at different levels (local, regional and national) as well as decision makers could benefit from advanced GIS applications which provide a toolbox for trend analyses, impact briefings and policy recommendations in order to reduce undernourishment and malnutrition in developing countries.
